# Changes in Triacylglycerols Content and Quality Control Implications of Coix Seeds during Processing and Storage

**DOI:** 10.3390/foods11162462

**Published:** 2022-08-16

**Authors:** Weiwei Tang, Jiancheng Wang, Wei Li, Chaojun Zhang, Ping Li, Jun Chen

**Affiliations:** 1 State Key Laboratory of Natural Medicines, China Pharmaceutical University, Nanjing 210009, China; 2 Zhejiang Kanglaite Pharmaceutical Co., Ltd., Hangzhou 310018, China; 3 Department of Pharmacognosy, School of Traditional Chinese Pharmacy, China Pharmaceutical University, Nanjing 211198, China

**Keywords:** coix seed, triacylglycerols, quality control, processing, storage

## Abstract

Coix seed is a kind of widespread cereal, and it is used as a folk medicine in China. The present work focuses on the analysis of changes in triacylglycerols (TAGs) content and mycotoxins of coix seed during the processing and storage period for its quality control. Using ultra-high-performance liquid chromatography coupled with quadrupole time-of-flight tandem mass spectrometry (UHPLC-Q-TOF-MS) and high-performance liquid chromatography coupled with evaporative light-scattering detector (HPLC-ELSD) techniques, 42 lipid components in coix seeds were identified, and seven molecular species of TAG in coix seeds from different localities in China were measured and compared, respectively. A correlation analysis between the morphological features and TAGs contents revealed the integrity instead of the particle size of the seed, displaying a highly positive correlation with its quality. The higher contents of TAGs in hulled coix seed than in polished coix seed proposed an alternative processing way. During storage, the changes in TAGs contents of seeds indicated that the storage period should be less than 3 months, and the intact seeds could maintain lipid stability better than the powder. Furthermore, the air humidity and temperature should be controlled during coix seed storage to prevent the production of mycotoxins. These results provide significant insight into the effective control of coix seed quality during processing and storage.

## 1. Introduction

Coix seed is the seed of *Coix lachryma-jobi* L. var. ma-yuen Stapf and also known as adlay, Job’s tears, Coicis Semen, Yiyiren. Coix seed is an important minor cereal with high nutritional and medicinal value [1,2]. It is widely used as food [3], an ingredient for brewing [4], nutraceuticals supplements [5] and for some adjuvant oncology therapies [6]. Lipids, especially triglycerides, are one of the main constituents of coix seed. Previous studies exhibited the lipid contents in polished coix seed ranging from 5.1 to 9.4% [7,8], which is significantly higher than in most common cereals. The highly abundant lipid contents in coix seed inspired the research to explore it for the quality control evaluation of coix seed. A previous study indicated that lipids could potentially be used as a key index to distinguish the geographical origin of coix seeds [9]. Triolein, one of the main triacylglycerols in coix seed, has been investigated and applied as a marker compound for quality control [7]. In addition, the total contents of triacylglycerols (TAGs) are widely used for quality control of coix seeds in most regulations. The US pharmacopeia required that a summation of seven TAGs contents should be more than 3.5% in coix seeds. Both Chinese and European pharmacopeia defined that the content of triolein should not be less than 0.5%. However, the current research mainly focuses on the highly abundant lipids, such as triolein, since there are barely any comprehensive reports related to global mapping lipids in coix seed. Therefore, an in-depth analysis of its lipid profiling should be characterized for a deeper understanding and more use in quality control of coix seed.

The production process of coix seeds generally involves drying, hulling, polishing and storage. During the harvest and processing procedures of coix seeds, the morphological features are first assessed. As described in the US pharmacopeia [10], the macroscopic characteristics, including the shape, color, size and texture, are primarily used for quality evaluation. The appearance of coix seed is one of the most important characteristics for its classification and grading concerning the commercial quality and organoleptic properties [11]. Global evaluation of the morphological features either in a metric or subjective manner is an important quality indicator throughout the whole commercial utilization chain, from the production to the storage, the marketing, and finally, down to the consumer. However, the integrity of coix seed has not been considered for its quality evaluation. During the harvest and processing of coix seeds, both manual and mechanical manipulation may cause the breakage of the seeds. It was supposed that the damage to each grain affects the loss of quality because the oxidation of the ingredients may begin.

Although both morphological features and TAGs content have been used for quality evaluation, the relationships between them have not been clarified yet. Moreover, other features of the seeds, such as the shell, bran and core, may exert influence on the product quality. During the processing, both the shell and the bran are removed during rice milling to produce the coix seed. All cereals tend to have lipids concentrated in the bran, including rice [12]. Thus, it is necessary to elucidate the role of bran in the quality control of coix seeds during processing. Except for processing, the harvested or processed coix seeds are normally stored to achieve a continued supply to the market. During storage, the physicochemical properties of coix seeds may change under the influence of internal and external factors. It is obvious that there are changes of TAGs content during storage, thus causing a decline in quality [13]. Lipids are known to be highly perishable because they are susceptible to oxidation and hydrolysis, which lead to the decomposition of ingredients and lost properties [14]. Therefore, the stability of the coix seeds under different storage times should be evaluated. Moreover, cereal foods could be easily contaminated by mycotoxins under storage due to the growth of some fungi [15]. Aflatoxins B1, B2, G1, G2, the most common toxics in coix seed, are produced by fungi belonging to *Aspergillus flavus*. Aflatoxins are known as group 1 carcinogenic compounds, which could be produced quickly under warm and moist conditions [16]. Zearalenone is another toxic compound commonly found in coix seed, which could cause severe adverse effects, such as hepatotoxicity, neurotoxicity and estrogenic effects [17]. Aflatoxins and zearalenone are considered stable compounds, which cannot be broken down through heating, cooking or brewing operations [18,19]. Thus, it is necessary to monitor the content of aflatoxins and zearalenone in coix seed during storage for safety.

In this study, the comprehensive lipid profiling of coix seeds was analyzed by UHPLC-QTOF-MS techniques. High-performance liquid chromatography coupled with evaporative light-scattering detector (HPLC-ELSD) method was applied to quantify seven TAGs contents to explore the factors that affect the quality of coix seeds during processing and storage. An immunoaffinity assay coupled with LC-MS/MS detection was used for monitoring the aflatoxins and zearalenone in the coix seed, thus providing more reliable suggestions for quality control.

## 2. Materials and Methods

### 2.1. Materials and Reagents

A total of 83 batches of coix seeds for the present study were collected in various production regions in China, including Guizhou, Yunnan, Zhejiang, Fujian and Jiangxi Provinces (Table S1). Trilinolein (LLL), 1,2-dilinoleoyl-3-palmitin (LLP), 1,2-dilinoleoyl-3-olein (LLO), 1-palmitoyl-2-oleoyl-3-linolein (POL), 1,2-dioleoyl-3-linolein (OOL), 1,2-dioleoyl-3-palmitin (OOP) and triolein (OOO) were used as reference compounds. OOO was obtained from Shanghai Aladdin Bio-Chem Technology Co., LTD (Shanghai, China), and other compounds were obtained from Tianjin NNU Biological Technology Co. Ltd. (Tianjin, China). The purity of each reference compound determined by HPLC-ELSD was above 98%. A Milli-Q water purification system from Millipore (Bedford, MA, USA) was utilized for purifying deionized water. Acetonitrile, methanol (MS-grade) and dichloromethane (HPLC grade) were obtained from Merck (Darmstadt, Germany). Formic acid (HPLC-grade) and ammonium formate (HPLC-grade) were obtained from ROE scientific Inc. (Newark, DE, USA) and Sigma-Aldrich, respectively. Standard solution of zearalenone (25 μg/mL) and mixed standard solutions of aflatoxins containing G2 (0.3 μg/mL), B2 (0.3 μg/mL), G1 (1.0 μg/mL) and B1 (1.0 μg/mL) were purchased from Supelco (Bellefonte, PA, USA). The immunoaffinity column kits containing antibodies to both aflatoxins and zearalenone were obtained from Beijing Zhong Ke Hui Ren Technology Co., Ltd. (Beijing, China).

### 2.2. Sample Preparation

The TAG reference was dissolved in the dichloromethane solvent first to prepare the stock solution. Then, the stock solution was dissolved in methanol to obtain a final concentration of 1 mg/mL. The stock solutions were diluted with methanol to prepare the desired working solutions of TAGs standards. For sample solution preparation, finely powdered coix seeds were prepared using the pulverizer grinding machine, screened through a No.4 sieve (250 μm). An aliquot of 500 mg powder was accurately weighed and then mixed with 50 mL methanol for ultrasonic extraction (200 W, 40 kHz) for 0.5 h at room temperature. After 30 min extraction, the mixture was cooled to room temperature, and methanol was added for lost weight and then filtered with a 0.45 μm filter. All solutions were stored at 4 °C until use. Samples for aflatoxins and zearalenone detection were prepared as follows: 3 g and 5 g of powdered coix seeds were weighed and dissolved in 15 mL of 70% methanol and 90% acetonitrile solutions, respectively. Both solutions were added to 0.6 g NaCl and then vortexed for 1 min. After centrifugation for 5 min under 2500 rpm/min, 5 mL of the supernatant was transferred and diluted to 25 mL with deionized water. Then, the samples were filtered with a 0.45 μm filter and performed on the immunoaffinity column, according to the kits’ instructions.

### 2.3. UHPLC-QTOF-MS Conditions

An Agilent 1290 series LC system coupled with an Agilent 6530 QTOF tandem mass spectrometer (Agilent Technologies, Santa Clara, CA, USA) was implemented for qualitative analysis. The LC system was equipped with an online degasser, a binary pump, an auto-sampler and a thermostatically controlled column compartment. The column oven temperature was set at 25 °C throughout the analytical process. Chromatographic separation was achieved with an Agilent Infinity Lab Poroshell120 EC-C8 column (3.0 mm × 100 mm, 2.7 μm). The mobile phases were water containing 5 mM ammonium formate (A) and methanol containing 5 mM ammonium formate (B). The gradient elution program was set to 15~70% B at 0~20 min, 70~80% B at 20~25 min, 80~100% B at 25~45 min and 100% B at 45~65 min. The flow rate was set at 0.3 mL/min, and the injection volume was 2 µL.

For MS and MS/MS results acquisition, the drying gas (N2) flow rate was set as 10 L/min at 350 °C, while the sheath gas flow rate was set as 11 L/min at 350 °C. Other parameters were set as follows: nebulizer, 35 psig; capillary voltages, 3000 V; fragmentor, 135 V; collision energy was set at 15 eV, 30 eV and 50 eV. The data were acquired under both positive and negative ion modes. The mass ranges were set at *m/z* 100–2000.

### 2.4. HPLC-ELSD Conditions and Single Standard to Determine Multi-Components (SSDMC) Method for Quantitation of TAGs

Quantitative analysis was carried out on a Shimadzu LC-20AT Series HPLC system equipped with PL-ELS 1000 evaporative light-scattering detector (Shimadzu, Kyoto, Japan), a quaternary solvent delivery system and a column temperature controller. Chromatographic separation was conducted with the Agilent Infinity Lab Poroshell120 EC-C8 column (2.7 μm, 3.0 mm × 100 mm). The mobile phase was methanol with isocratic elution, and the flow rate was 0.3 mL/min. The column oven temperature was set at 25 °C throughout the analytical process, and the injection volume was 5 µL. The ELSD detector parameters were set as follows: nebulizing gas pressure, 3.4 bar; heating tube temperature, 40 °C; gain value, 11; filter value, 10 s. Shimadzu LC solution software (ver. 1.21SPI) was used for data collection.

For the quantitation of TAGs, the SSDMC method was developed according to a previous study [7]. The OOO was selected as the internal single standard, and the relative conversion factor (RCF, Fi) was displayed in Table S2. Briefly, the RCF was calculated based on the correlation between the chromatographic response and the concentration of the analyte. Due to the similar structures of these seven compounds, the RCFs of these six analytes compared to OOO were finally confirmed as 1.0.

### 2.5. UHPLC-QQQ-MS Conditions for the Analysis of Aflatoxins and Zearalenone

A Shimadzu LC-30A UHPLC combined with a Shimadzu 8050 QQQ MS (Kyoto, Japan) were used for detection. An Agilent ZORBAX SB-C18 (4.6 × 50 mm, 1.8 μm) column was used for chromatographic separation. The mobile phases were 0.1% formic acid water solution containing 10 mM ammonium acetate (A), acetonitrile (B), water (C) and methanol (D). The liquid chromatographical conditions were set as follows: for aflatoxins, mobile phases A and B were used, and the elution program was set as 0~4.5 min, 35~85% B; 4.5~6 min, 85~100% B; 6~6.5 min, 100~35% B; 6.5~10 min, 35% B at a column temperature of 25 °C. For zearalenone, mobile phases C and D were used, and the elution program was set as 0~5 min, 25~75% D; 5~6 min, 70% D; 6~9 min, 70~25% D at a column temperature of 40 °C. The flow rate and injection volume were set as 0.3 mL/min and 1.0 µL, respectively, for both aflatoxins and zearalenone.

The QQQ-MS were equipped with an ESI source and operated with the following parameters: interface voltage, 5500 V; nebulizer gas (N2) flow rate, 2.0 L/min; drying gas flow rate, 10.0 L/min. The interface temperatures were set as 550 °C and 350 °C for aflatoxins and zearalenone, respectively. The multiple reaction monitoring (MRM) mode was used for quantification, and the parameters are displayed in Table 1.

### 2.6. Data Analysis

Agilent MassHunter Qualitative Analysis Software (Version B.07.00) was used for qualitative analysis. Graphpad prism 8.0 and Microsoft Excel 2019 were utilized for data analysis. Shimadzu LC solution software (ver. 1.21SPI) was used for quantitation. For LC-MS/MS quantitation, the software of Shimadzu Labsolutions LCMS Version 5.65 (Kyoto, Japan) was used.

## 3. Results and Discussion

### 3.1. Overall Lipid Profiling of Coix Seeds

**Figure 1:**
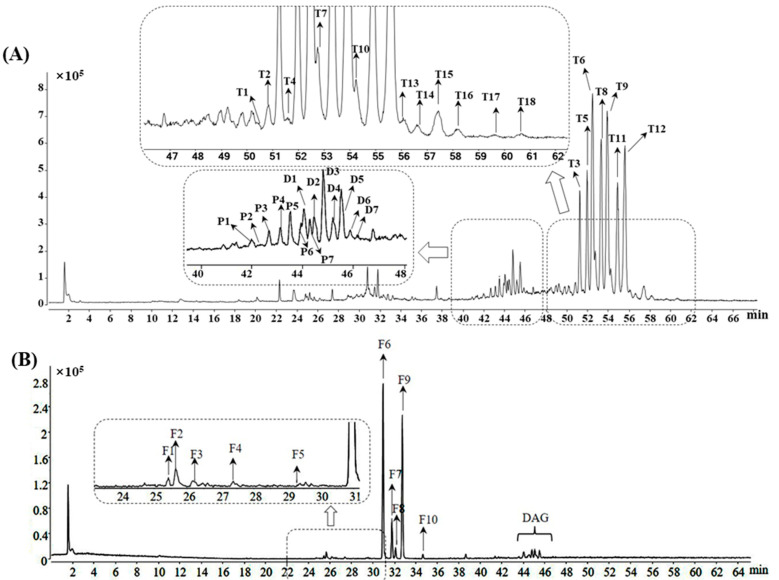
Total ion chromatogram of coix seed sample under positive (**A**) and negative (**B**) detection mode by UHPLC-QTOF-MS.

Lipids, especially TAGs and diacylglycerols (DAGs), are considered some of the main constituents of coix seeds [20]. In this study, the comprehensive lipid features of coix seeds, including TAGs, DAGs, phosphatidylcholines (PCs) and free fatty acids (FAs), were profiled using the UHPLC-QTOF-MS method under the positive and negative ion modes. Based on the analysis from the MS total ion chromatogram, a total of 32 peaks and 10 peaks were detected under positive and negative ion modes, respectively (shown in Figure 1A). Among these peaks, seven components were unambiguously authenticated as LLL (T3), LLP (T5), LLO (T6), POL (T8), OOL (T9), OOP (T11) and OOO (T12) by comparing their retention times, *m/z* values and fragment ions with those of the reference compounds. In addition to the above 7 compounds, 16 other compounds (Table S3) were tentatively identified as TAGs by comparison with the MS and MS/MS spectrometry features of the compounds from previous literature works [9,21,22]. Since the molecular structure of TAG contains three acyl chains, the ions of the fatty acyl group and the residues after the eliminated acyl chain were generated under MS/MS detection [23]. The majority of the fatty acyl in coix seeds is unsaturated, thus making palmitic-, stearic-, oleic- and linoleic acyl-triglycerides the main components of lipids [24]. Using LLP (T5) as an example, the typical [M + NH_4_]+ adduct ion with *m/z* 872.7729 was observed in MS spectrometry owing to the non-polar nature of TAGs. Through the collision-induced dissociation (CID) procedure, the MS/MS pattern was acquired. The typical [M + NH_4_]+ adduct ion produces abundant fatty-acyl-specific fragment ions (as combined loss of NH3 and fatty acyl), allowing the characterization of TAGs structures. As shown in Table S3, the *m/z* 263.2369 in the product ions list was characterized as the acyl chain of linoleic acid [C18H32O2-OH] fragmented from LLP. In addition, the MS/MS product ions at *m/z* 599.5045, *m/z* 575.5042, *m/z* 337.2751 and *m/z* 313.2714 are derived from the moiety after the loss of acyl chains corresponding to [M + H-palmitic acyl]+, [M + H-linoleic acyl]+, [M + H-palmitic acyl-linoleic acyl + H2O]+ and [M + H-2linoleic acyl + H2O]+, respectively (shown in Figure S1). DAGs were also detected in coix seeds in this study. As shown in Table S4, the retention times of DAGs (marked as D1 to D7) were shorter than TAGs due to their higher polarity. For MS spectrometry, the quasi-molecular ion peaks of DAGs were different from those of TAGs, with the adduct ion of [M + Na]+ easily generated (shown in Table S4). Due to the similar molecular structures, it is obvious that the fragment pathway of TAGs and DAGs should be alike. From TAGs, the MS/MS product ions of the acyl chain and the moiety after the loss of the acyl chain were fragmented easily and could be applied to the identification of DAGs (Figure S1 showed the typical fragment pathway of DAG:LP). Moreover, seven molecular species of PC were also characterized in this study. As shown in Table S5, the retention times of PCs were less than DAGs (marked as P1 to P7) due to the higher polarity of the phosphorylcholine group. The quasi-molecular ion peaks of PCs were displayed as [M + H]+ adduct ions (Table S5). For the fragmentation of PCs, the product ions of the acyl chain or the moiety after the loss of the acyl chain could be characterized and assigned for the identification of the composition of PCs. More importantly, the presence of m/z 184, 86 and 60 was recognized as the MS/MS product ions of the phosphatidylcholine group (as shown in Figure S1), thus providing distinct diagnostic ions for the identification of PCs.

As for the negative mode detection, linoleic acid (F6), palmitic acid (F7), oleic acid (F9) and stearic acid (F10) were elucidated by comparing the MS data with those of the reference compounds (Figure 1B and Table S6). Another six fatty acids were also characterized according to the previous data [25]. Although gas chromatography (GC) was the most commonly used technique for fatty acids analysis, the HPLC-QTOF/MS technique was considered as an alternative assay, as previously reported [26]. Among the peaks assigned as fatty acids in this study, F1, F2 and F3 were detected with the same *m/z* 295 but at different retention times (25.4, 25.6 and 26.1 min, respectively). Compared with the previous report, these three peaks were tentatively proposed as the hydroxy octadecadienoic acid and its isomers with different locations of the hydroxy group or double bonds. Overall, a total of 42 lipids were identified in this study, thus giving a better understanding of the chemical profile of coix seeds.

### 3.2. Quantitation Analysis of Seven TAGs in Coix Seeds

Lipids, among others, are the main active constituents, and according to the above chemical profile, TAGs are the primary constituents of coix seeds. According to previous studies [7,9], seven TAGs, namely LLL (T3), LLP (T5), LLO (T6), POL (T8), OOL (T9), OOP (T11) and OOO (T12), were selected as the mark components of coix seeds. Similarly, due to the high content of OOO in coix seeds and its commercial standard products available, it has been used as the compound mark of coix seeds in Chinese and British pharmacopeias. Moreover, the total content of these seven TAGs is used for quality control in US pharmacopeia. In this study, the SSDMC method using OOO as a reference compound was applied for the quantification of these seven TAGs by using the HPLC-ELSD technique.

**Figure 2:**
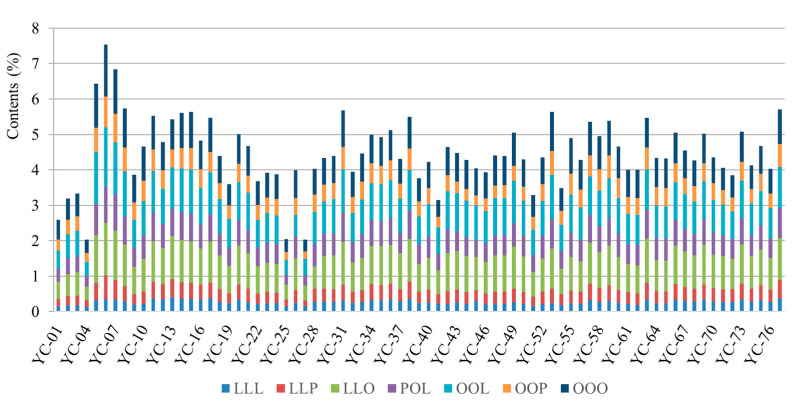
The seven molecular species of TAGs from 77 batches of coix seed (from YC-01 to YC-77). Each color represents different analytes.

A total of 77 batches of coix seed from different geographic areas, namely Fujian, Yunnan, Guizhou, Zhejiang and Jiangxi provinces, were investigated using the SSDMC method. The contents (%) for the percentage of TAGs among the original weight of coix seed were calculated. As shown in Figure 2 and Table S7, OOO, OOL and LLO were displayed as high content, corresponding to the range of 0.32–1.47%, 0.41–1.67% and 0.38–1.47%. The average amount of LLL, LLP, LLO, POL, OOL, OOP, OOO as total TAG was calculated as 5.97%, 8.36%, 21.24%, 14.2%, 21.6%, 10.72%, 17.9%, respectively. A previous study [7] revealed that oleic acid and linolenic acid were the most abundant FAs in coix seed. The TAGs with oleic acid and linolenic acid components were also in high concentration. As one of the most abundant compounds, OOO was marked as a key component required at more than 0.5% in coix seed by the 2020 edition Chinese pharmacopoeia. The results indicated that 94.8% of the 77 batches of coix seed achieved this quality standard. In addition, the total contents of these seven TAGs varied from 2.0 to 7.5% in coix seeds. According to the US pharmacopeia, the total content of TAGs should be more than 3.5%. According to this stated specification, 88.3% of the 77 batches of coix seed qualified in this study.

### 3.3. Evaluation of the Correlations between the Morphological Features of Coix Seed and Its Quality

**Figure 3:**
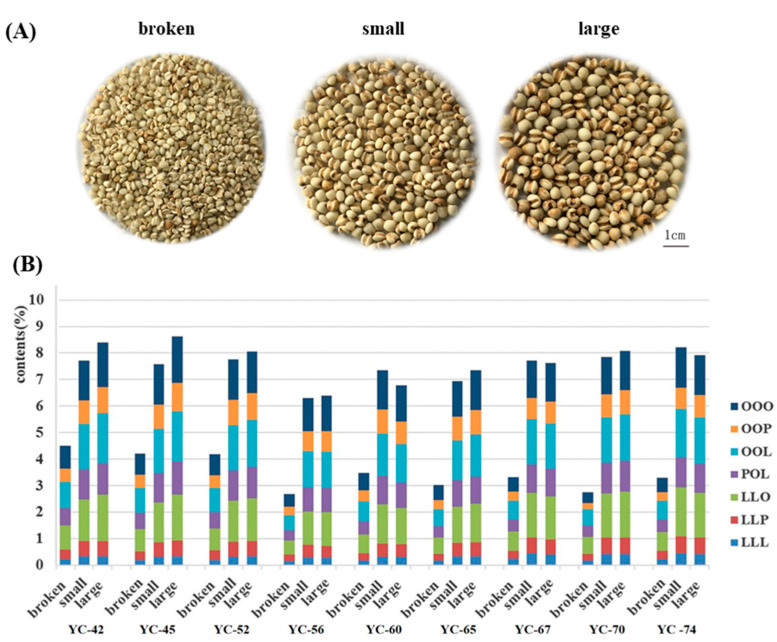
The optical graphs (**A**) and the content of TAGs (**B**) from the broken grain, small grain (width < 4.5 mm) and large grain (width > 5.5 mm).

The traditional classification of the coix seed depended on its morphological characteristics. During harvest and manufacturing, the morphological features of coix seeds are primarily evaluated for their quality. Those features, such as larger width and full shape of the coix seed, were considered as good quality. However, whether it is reasonable to evaluate the quality of coix seed using these morphological features has not been elucidated scientifically. Therefore, the relationship between the content of these seven TAGs and the morphological characteristics was explored in this section. Since the coix seed with broken features may be selected or used during processing, the integrity of the coix seeds has also been considered. A total of nine batches of coix seed as described above were used for the determination of width, 1000-grain weight, integrity and the content of TAGs. All data were used for the correlation analysis with SPSS Statistics 22 Software (IBM, Chicago, IL, USA). As shown in Table 2, 1000-grain weight displayed high correlations with width (correlation coefficient r^2^ = 0.439), and integrity showed high correlations with TAGs content (correlation coefficient r^2^ = 0.558). However, neither the width of the coix seed nor the 1000-grain weight displayed poor correlation with the content of TAGs (correlation coefficient r^2^ = 0.006 and 0.129, respectively). The results indicated that the width of the coix seed does not affect its quality. Significantly, the integrity of the coix seed may affect the contents of TAGs. To further validate this, samples from the same batches were used. Samples from Guizhou province (YC-42, 45, 52), Yunnan province (YC-56, 60, 65) and Fujian province (YC-67, 70, 74) were categorized into three groups: the broken grain, the small grain with a width of less than 4.5 mm and the large grain with a width over 5.5 mm (Figure 3A). These three groups were divided through suitable sieving and vernier caliper measurement. Using SSDMC for TAGs determination, the results (as shown in Figure 3B) of the total TAGs in broken grains were lower and significantly different to the large and small grain (*p* < 0.001). The result displayed a total TAGs content of over 6.0% for the small and large samples but below 5.0% or even 3.0% for those broken samples. Moreover, the content of each TAGs (namely OOO, OOP, OOL, LLO, LLP, LLL) in the broken, large and small grain also showed a similar trend. The contents of these seven TAGs for small and large seeds were 1.5- to 2.0-fold higher than those of the broken samples. However, there was no significant difference in the content of total TAGs or each TAGs between the large grain and small grain (*p* = 0.792). Previous studies had indicated that the size of the coix seed was considered primarily as a quality control parameter for sample collection and processing. Using traditional morphological features for evaluation, those broken samples but ones with a suitable size would have been processed. Nevertheless, as the results depicted, the broken grains may cause a decrease in the TAGs content, thus providing worse quality and poor yield of TAGs in the final product. Therefore, the integrity of the seed should be taken into consideration for better quality control, and the standardization process of the coix seed should be intensively evaluated to guarantee the quality consistency.

**Figure 4:**
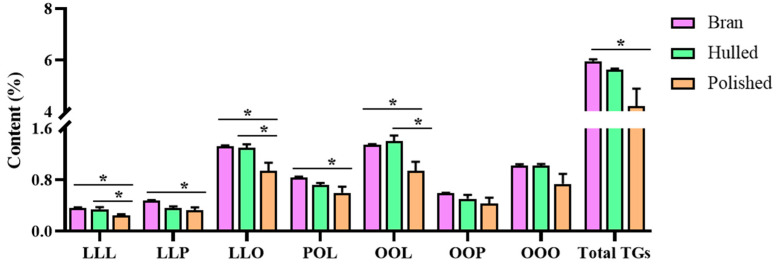
Contents of triglycerides from the hulled, polished coix seed and the bran, * *p* < 0.05.

The coix seed is a ripe caryopsis with a hierarchical structure of the shell, bran and kernel. During processing, the dried coix seed is freed from the shell and then polished to collect the kernel articles. To provide a reasonable basis for processing, the TAGs contents in the different parts of the coix seed were investigated in this section. Three batches of the coix seed (YC-78, YC-79 and YC-80) were acquired and processed with and without the shell. The samples were collected separately and divided into three groups (the hulled coix seed group, the polished coix seed group and the coix seed bran group) for the TAGs content determination. As shown in Figure 4, both the total and each TAGs in the coix seed bran exhibited no significant difference compared with the hulled coix seed. However, the content of TAGs was lower in the polished coix seed than in these two forms. As noted before, the bran usually contains the bulk of the lipids. During processing, the hulled coix seeds are polished to remove the bran. From the quantitation results, the bran appears valuable for obtaining the TAGs and could be preserved during processing. Previous studies have reported that the bran of coix seed exhibited many pharmacological effects, including anti-inflammatory [27,28,29,30], thus indicating the importance of the bran. It is therefore recommended that the bran be optional, but the integrity should be maintained during processing to make the quality more controllable.

### 3.4. Quality Control of Coix Seed during Storage

**Figure 5:**
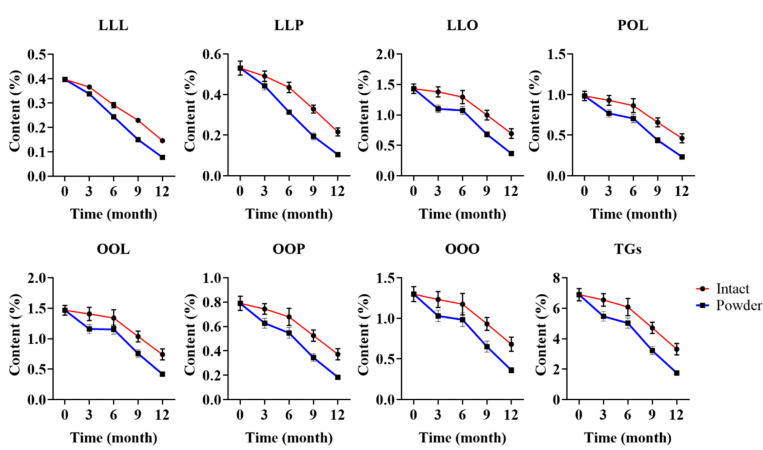
Changes of TAGs content under long-term storage.

For large-scale production, a lot of coix seed samples are collected and stored until processing. The long-term stability research of the TAGs content is critical for the quality evaluation during the sample storage. A previous study [13] established changes of coix seed under storage. To evaluate the changes of TAGs content, three batch samples (YC-81, YC-82 and YC-83) were collected and divided into two groups (intact coix seed and coix seed powder) and then stored in a cool and dry place at room temperature. Each sample was then evaluated at three months interval for one year. As shown in Figure 5, both the total and each TAGs content showed little change in the first three months in the intact coix seed. As shown in Table S8, the decline rate of the total TAGs content was in the range from 2.03% to 8.06%. After six months, the decline rate was still below 15%. However, the situation was different in the coix seed powder, which exhibited a 20% decline rate in the first three months. The results in Figure 5 demonstrate that the contents of OOO and total TAGs were below 0.5% and 3.5% in the coix seed powder after 9 months of storage, respectively, while those of the intact coix seed were not. After 12 months of storage, the total TAGs in both the intact coix seed and the coix seed powder were below 50% of the original content. From the result, we suggest that the storage time of coix seed should be less than 3 months for better quality control. In addition, the intact coix seed was shown to be more stable than the coix seed powder, which implied that the bran played an important role in maintaining the integrity of the TAGs.

Similar to other edible grains, coix seed was easily contaminated by aflatoxins and zearalenone due to improper storage conditions. To analyze the trace amounts of aflatoxins and zearalenone in the coix seed, the immunoaffinity column was used for the extraction of analytes and then coupled with the LC-MS/MS technique for detection, according to previous studies [31,32]. The sensitivity and linearity for the detection of aflatoxin G2, G1, B2, B1 and zearalenone are displayed in Table S9. This method was applied to measure the content of aflatoxins and zearalenone in 24 batches of coix seed. As displayed in Table S10, all batches of coix seed were regarded as negative samples of aflatoxin G2 and G1, which were low in coix seed that exceeded the LOD of this study. As for aflatoxin B2 and B1, 21 and 20 batches were regarded as negative samples, respectively. For zearalenone, all samples were shown to be positively detected in the range of 2.31–114.58 μg/kg. According to the China national food safety standards (GB 5009.209-2016), the limit of zearalenone should be below 60 μg/kg in cereal and relevant products. Only one batch of coix seed exceeded this content. To further investigate the changes of aflatoxins and zearalenone under different storage conditions, temperatures ranging from 4 °C to 35 °C and different relative humidity ranges from 50% to 97% were adopted in this study. The storage time was set at four weeks. As displayed in Table 3, aflatoxins G2, G1 and B2 were negatively detected under different conditions. However, 0.61 μg/kg of aflatoxin B1 was detected when the condition changed to 25 °C with 97% humidity. As for zearalenone, it was obviously increased as the temperature and humidity increased. The results reveal that the content of zearalenone was easily affected by humidity, as it was increased over 60 μg/kg when the humidity increased up to 90%. These results suggested that the temperature and humidity should be controlled during storage.

## 4. Conclusions

In this work, the comprehensive lipid profiling of the coix seed was tentatively characterized by the UHPLC-Q-TOF-MS/MS technique. A total of 42 lipids, thus 18 triglycerides, 7 diglycerides, 7 phospholipids and 10 fatty acids, were identified, hence providing a better understanding of the chemical profiles of the coix seed. For quality control, seven components of TAGs were quantified in this study, especially during the processing and storage procedures of the coix seed. The information from the results indicated that integrity, instead of size, displayed a close correlation with the content of TAGs. The positive correlation between the integrity and content may be useful for assessing the quality during the collection and processing of the coix seed. The quantitative analysis of TAGs from different parts of the coix seed implied that bran was important and could be maintained during processing. The long-term stability research of the TAGs content proved that triglycerides in the integral coix seed were more stable than those in powdered coix seeds. Similarly, a shorter storage time of less than 3 months does not greatly affect the product quality. Moreover, the temperature and humidity of storage conditions should be strictly controlled to prevent the production of toxic secondary metabolites of fungi. The results obtained from this study would help establish a scientific and rational quality control strategy for the coix seed, especially in the procedures of processing and storage.

## Figures and Tables

**Table 1:** MS/MS parameters for aflatoxins and zearalenone detection.

Analyte	Parent Ion	Product Ion	CE (eV)
Aflatoxin G2	[M + H] + 331.1	**313.1**	33
		245.1	40
Aflatoxin G1	[M + H] + 329.1	**243.1**	35
		311.1	30
Aflatoxin B2	[M + H] + 315.1	**259.1**	35
		287.1	40
Aflatoxin B1	[M + H] + 313.1	241	50
		**285.1**	40
Zearalenone	[M − H] − 317.1	**174.9**	25

bold ions were used for quantitation analysis.

**Table 2:** Correlation analysis between 1000-grain weight, integrity, width, triglycerides.

Correlation	1000-Grain Weight	Integrity	Width	Triglycerides
1000-grain weight		0.104	0.439 **	0.129
integrity			−0.039	0.558 **
width				0.066
triglycerides				

** Significant difference (*p* < 0.01).

**Table 3:** Changes of aflatoxins and zearalenone under different storage conditions.

Temperature/Relative Humidity (%)	4 °C/50	25 °C/50	35 °C/50	25 °C/97	25 °C/90	25 °C/81
Aflatoxin G2 (μg/kg)	ND	ND	ND	ND	ND	ND
AflatoxinG1 (μg/kg)	ND	ND	ND	ND	ND	ND
AflatoxinB2 (μg/kg)	ND	ND	ND	ND	ND	ND
AflatoxinB1 (μg/kg)	ND	ND	ND	0.61	ND	ND
Zearalenone (μg/kg)	2.61	4.93	30.25	91.71	54.62	81.92

## Data Availability

All of the data is contained within the article and the Supplementary Materials.

## Supplementary Materials

The following supporting information can be downloaded at: https://www.mdpi.com/article/10.3390/foods11162462/s1, Table S1: Geographical origins of 83 batches of coix seed; Table S2: The conversion factors of the seven analytes; Table S3: Triacylglycerols identified in coix seed by UHPLC-Q-TOF-MS; Table S4: Diacylglycerols identified in coix seed by UHPLC-Q-TOF-MS; Table S5: Phosphatidylcholines identified in coix seed by UHPLC-Q-TOF-MS; Table S6: Fatty acids identified in coix seed by UHPLC-Q-TOF-MS; Figure S1: Fragmentation pattern of lipid compounds; Table S7: The seven molecular species of TG contents from 77 batches of coix seed (from YC-01 to YC-77); Table S8: The rate of decline in the content of coix seed and coix seed powder; Table S9: The calibration curves, linear range, limit of detection (LOD) and limit of quantification (LOQ) of the aflatoxins and zearalenone; Table S10: Contents of aflatoxin and zearalenone in 24 batches of coix seed.
