# Supplementary material for: Changes in Triacylglycerols Content and Quality Control Implications of Coix Seeds during Processing and Storage
Source: Foods. 2022 Aug 16;11(16):2462. doi: 10.3390/foods11162462 (PMC9407508; doi:10.3390/foods11162462)
Supplement: Supplementary file 1 [file foods-11-02462-s001.zip › foods-1825741-supplementary.pdf]

Supplementary materials

Changes in triacylglycerols content and quality control implications of Coix Seeds during processing and storage

Weiwei Tang[a,#], Jiancheng Wang[a,#], Wei Li[a], Chaojun Zhang[b], Bin Li[a], Ping Li[a,]*, Jun Chen[c,]*

[a] *State Key Laboratory of Natural Medicines, China Pharmaceutical University, Nanjing, Jiangsu 210009, P. R. China*

[b]*Zhejiang Kanglaite Pharmaceutical Co. Ltd, Hangzhou, Zhejiang 310018, P. R. China*

[c]*Department of Pharmacognosy, School of Traditional Chinese Pharmacy, China Pharmaceutical University, Nanjing, Jiangsu 211198, P. R. China*

*Correspondence: liping@cpu.edu.cn (P.L.); 1020060894@cpu.edu.cn (J.C.)

[#]These authors contributed equally to this work.

Content

Table S1: Geographical origins of 83 batches of coix seed;

Table S2: The conversion factors of the seven analytes;

Table S3: Triacylglycerols identified in coix seed by UHPLC-Q-TOF-MS;

Table S4: Diacylglycerols identified in coix seed by UHPLC-Q-TOF-MS;

Table S5: Phosphatidylcholines identified in coix seed by UHPLC-Q-TOF-MS;

Table S6: Fatty acids identified in coix seed by UHPLC-Q-TOF-MS;

Figure S1: Fragmentation pattern of lipids compounds;

Table S7: The seven molecular species of TG contents from 77 batches of coix seed (from YC-01 to YC-77);

Table S8: The rate of decline in the content of coix seed and coix seed powder;

Table S9: The calibration curves, linear range, limit of detection (LOD) and limit of quantification (LOQ) of the aflatoxins and zearalenone;

Table S10: Contents of aflatoxin and zearalenone in 24 batches of coix seed.

Table S1 Geographical origins of 83 batches of coix seed

No.	Origins	No.	Origins	No.	Origins
YC-01	Huilong, Guizhou	YC-29	Yaozhai, Fujian	YC-57	Lugu, Yunnan
YC-02	Xiashan, Guizhou	YC-30	Dongkeng, Fujian	YC-58	Bangbie, Yunnan
YC-03	Baling, Guizhou	YC-31	Lichu, Fujian	YC-59	Sheli, Yunnan
YC-04	Tunjiao, Guizhou	YC-32	Miaowan, Fujian	YC-60	Xinzhu, Yunnan
YC-05	Lehe, Guizhou	YC-33	Maochu, Fujian	YC-61	Lubuli, Yunnan
YC-06	Sanhe, Guizhou	YC-34	Yuanling, Fujian	YC-62	Bianliao, Yunnan
YC-07	Poliu, Guizhou	YC-35	Dongyang, Fujian	YC-63	Kebai, Yunnan
YC-08	Longchang, Guizhou	YC-36	Hecun, Fujian	YC-64	Goujie, Yunnan
YC-09	Gaoliang, Yunnan	YC-37	Lichu, Fujian	YC-65	Niuwei, Yunnan

YC-10	Wulong, Yunnan	YC-38	Jinfengcun, Fujian	YC-66	Cunqiao, Fujian
YC-11	Dongzhuang, Zhejiang	YC-39	Nongchang, Jiangxi	YC-67	Yantang, Fujian
YC-12	Liuzhai, Zhejiang	YC-40	Liaohua, Jiangxi	YC-68	Meikeng, Fujian
YC-13	Xiaochi, Zhejiang	YC-41	Zhangqing, Jiangxi	YC-69	Jiuguan, Fujian
YC-14	Zhouling, Zhejiang	YC-42	Shizi, Guizhou	YC-70	Wangcun, Fujian
YC-15	Wengdi, Zhejiang	YC-43	Labi, Guizhou	YC-71	Dongyang, Fujian
YC-16	Houzhanggang, Zhejiang	YC-44	Ganlongdong, Guizhou	YC-72	Lichu, Fujian
YC-17	Yanshan, Zhejiang	YC-45	Xintian, Guizhou	YC-73	Yaozhai, Fujian
YC-18	Cangyang, Zhejiang	YC-46	Dapu,Guizhou	YC-74	Maochu, Fujian
YC-19	Shuangsheng, Zhejiang	YC-47	Xiaopingzhai, Guizhou	YC-75	Hecun, Fujian
YC-20	Tulin, Zhejiang	YC-48	Dianmu, Guizhou	YC-76	Lishi, Fujian
YC-21	Xinpan, Zhejiang	YC-49	Bashang, Guizhou	YC-77	Lingling, Fujian
YC-22	Wangcun, Fujian	YC-50	Shuanglong, Guizhou	YC-78	Taishun, Zhejiang
YC-23	Gaomen, Fujian	YC-51	Yangzhai, Guizhou	YC-79	Pucheng, Fujian
YC-24	Jiumu, Fujian	YC-52	Bangjie, Guizhou	YC-80	Shizong, Yunnan
YC-25	Huayuan, Fujian	YC-53	Huangtula, Guizhou	YC-81	Dongkeng, Fujian
YC-26	Zhongxinyuanli, Fujian	YC-54	Bianlin, Yunnan	YC-82	Hecun, Fujian
YC-27	Guanlu, Fujian	YC-55	Tianfang, Yunnan	YC-83	Yaozhai, Fujian
YC-28	Shipo, Fujian	YC-56	Yuxua, Yunnan		

Table S2 The conversion factors of the seven analytes

Analytes	*Fi*	RSD(%)
LLL	0.94	1.58
LLP	0.97	1.38
LLO	1.04	0.35
POL	0.95	1.38
OOL	1.06	0.60
OOP	0.97	0.91
OOO	1	0.60

Table S3 Triacylglycerols identified in coix seed by UHPLC-Q-TOF-MS.

No.	RT (min)	Precusor ion(m/z) $[M+NH_4]^+$	Molecular formula	Error (ppm)	MS/MS fragmentation (m/z)	Identification
T1	50.563	894.7563	$C_{57}H_{96}O_6$	-2.03	877.7286, 599.5057, 597.4910, 337.2781, 261.2186	TAG:LLLn
T2	50.995	870.7578	$C_{55}H_{96}O_6$	-3.85	853.7187, 597.4897, 573.4859, 337.2687, 313.2728, 261.9244	TAG:LLnP
T3*	51.297	896.7734	$C_{57}H_{98}O_6$	-3.68	879.7475, 861.7322, 599.5047, 337.2747, 319.2451, 263.2371, 245.2094	TAG:LLL
T4	51.887	846.7574	$C_{53}H_{96}O_6$	-3.84	573.4862, 549.4857	TAG:PoPL
T5*	52.015	872.7729	$C_{55}H_{98}O_6$	-3.2	855.7456, 599.5043, 575.5042, 337.2751, 313.2714, 263.2369	TAG:LLP
T6*	52.515	898.7892	$C_{57}H_{100}O_6$	-3.84	881.7589, 601.5191, 599.5047, 339.2886, 337.2746, 263.2375, 245.2263	TAG:LLO
T7	52.799	848.7724	$C_{53}H_{98}O_6$	-2.69	831.7462, 575.5047, 551.5041, 337.2790, 313.2732, 263.2362	TAG:PPL
T8*	53.349	874.7888	$C_{55}H_{100}O_6$	-3.48	857.7642, 601.5193, 577.5182, 575.5044, 339.2877, 313.2742, 263.2381, 239.2346	TAG:POL
T9*	53.917	900.8043	$C_{57}H_{102}O_6$	-3.21	883.7754, 603.5331, 601.5188, 339.2895, 263.2355, 245.2261	TAG:OOL
T10	54.284	850.7877	$C_{53}H_{100}O_6$	-3.34	833.7562, 577.5194, 551.5038, 339.2928, 313.2752, 265.2576, 239.2388	TAG:PPO
T11*	54.984	876.8042	$C_{55}H_{102}O_6$	-3.18	859.7768, 603.5349, 577.5190, 339.2891, 313.2772, 265.2522, 239.2362	TAG:OOP
T12*	55.652	902.8211	$C_{57}H_{104}O_6$	-4.5	885.7879, 603.5342, 339.2891, 321.2786, 265.2513, 247.2411	TAG:OOO
T13	56.152	928.8340	$C_{59}H_{106}O_6$	-1.35	911.7728, 631.5640, 599.5117, 369.3368, 337.2676, 263.2216, 265.2576	TAG:ALL
T14	56.636	878.8193	$C_{55}H_{104}O_6$	-2.54	861.7831, 605.5456, 579.5308, 577.5141, 341.3021, 339.2894, 313.2613, 267.2752, 265.2478, 239.1341	TAG:POS
T15	57.453	904.8352	$C_{57}H_{106}O_6$	-2.74	887.7946, 631.5722, 605.5434, 575.5054, 339.2891, 265.2490	TAG:POA
T16	58.288	930.8506	$C_{59}H_{108}O_6$	-2.39	913.7981, 631.5649, 601.5188, 369.3368, 339.2891, 263.2375	TAG:ALO

T17	59.706	906.8484	$C_{57}H_{108}O_6$	0.02	633.5834, 607.5769, 577.5260, 369.3369, 339.2891, 313.2631, 265.2478	TAG:AOP
T18	60.840	932.8618	$C_{59}H_{110}O_6$	-0.36	633.5783, 603.5311, 369.3318, 339.2891, 265.2478	TAG:AOO

A: arachidic acyl; Po:palmitoleic acyl; Rn:ricinoleic acyl; Ln:Linolenic acyl; L:linoleic acyl; O:oleic acyl; P:palmitic acyl; S:stearic acyl; TAG: LLP indicates that the three fatty acyl chains of triacylglycerol are linoleic acyl, linoleic acyl, and palmitic acyl respectively. * Structures confirmed by comparison with the reference standards.

Table S4 Diacylglycerols identified in coix seed by UHPLC-Q-TOF-MS.

No.	RT (min)	Precusor ion m/z [M+Na]$^+$	Molecular formula	Error (ppm)	MS/MS fragmentation	Identification
D1	44.195	639.4975	$C_{39}H_{68}O_5$	-2.92	617.5152, 599.5016, 337.2727, 263.2364	DAG:LL
D2	44.472	615.4967	$C_{37}H_{68}O_5$	-3.71	593.5151, 575.5066, 337.2734, 313.2735, 263.2358	DAG:LP
D3	44.858	641.5127	$C_{39}H_{70}O_5$	-4.44	619.5305, 601.5201, 339.2898, 337.2743, 263.2408	DAG:OL
D4	45.258	617.5132	$C_{37}H_{70}O_5$	-2.77	595.5309, 577.5202, 339.2833, 313.2776	DAG:PO
D5	45.575	643.5274	$C_{39}H_{72}O_5$	-2.74	621.5503, 603.5343, 339.2898, 265.2537	DAG:OO
D6	45.925	619.5286	$C_{37}H_{72}O_5$	-1.38	313.2668	DAG:PS
D7	46.157	645.5458	$C_{39}H_{74}O_5$	-2.97	341.3038	DAG:OS

DAG:PL indicates that the two fatty acyl chains of the diacylglycerol are palmitic acyl and linoleic acyl, respectively.

Table S5 Phosphatidylcholines identified in coix seed by UHPLC-Q-TOF-MS.

No.	RT (min)	Precusor ion m/z [M+H]$^+$	Molecular formular	Error (ppm)	MS/MS fragmentation	Identification
P1	41.995	780.5562	$C_{44}H_{78}NO_8P$	-2.92	597.4807, 520.3347, 502.3081, 184.0721, 166.0660, 125.9858	PC: LLn
P2	42.506	756.5557	$C_{42}H_{78}NO_8P$	-3.71	496.3363, 478.3266, 184.0725, 166.0654, 125.9836	PC: PLn
P3	42.689	782.5721	$C_{44}H_{80}NO_8P$	-4.44	559.4995, 520.3348, 502.3234, 184.0729, 166.0631, 125.9868	PC: LL

P4	43.122	758.5722	$C_{42}H_{80}NO_8P$	-2.77	575.5285, 520.3342, 502.3276, 496.3369, 478.3248, 184.0754, 125.9758	PC: LP
P5	43.523	784.5883	$C_{44}H_{82}NO_8P$	-2.74	601.5093, 522.3549, 520.3324, 502.2083, 184.0733, 166.0612, 125.9814	PC: OL
P6	44.090	760.5884	$C_{42}H_{83}NO_8P$	-1.38	577.5055, 504.3357, 496.3372, 478.3271, 184.0718, 166.0664, 125.9834	PC: PO
P7	44.290	786.6043	$C_{44}H_{84}NO_8P$	-2.97	603.5633, 524.3882, 520.3365, 502.3224, 184.0725, 166.0674, 125.9852	PC: SL

PL: LP indicates that the two fatty acyl chains of the phosphatidylcholine are palmitic acyl and linoleic acyl, respectively.

Table S6 Fatty acids identified in coix seed by UHPLC-Q-TOF-MS.

No.	RT (min)	Identification	Molecular formular	Precusor ion m/z $[M-H]^-$	Error (ppm)
F1	25.411	FA:C18:2 ,OH	$C_{18}H_{32}O_3$	295.2270	2.93
F2	25.661	FA:C18:2 ,OH	$C_{18}H_{32}O_3$	295.2285	-2.13
F3	26.178	FA:C18:2 ,OH	$C_{18}H_{32}O_3$	295.2391	-4.16
F4	27.363	FA:Rn	$C_{18}H_{34}O_3$	297.2473	3.75
F5	29.348	FA:Ln	$C_{18}H_{30}O_2$	277.2176	-1.06
F6*	30.966	FA:L	$C_{18}H_{32}O_2$	279.2368	-3.38
F7*	31.784	FA:P	$C_{16}H_{32}O_2$	255.2341	-4.47
F8	32.134	FA:B	$C_{22}H_{44}O_2$	339.2380	-8.95
F9*	32.735	FA:O	$C_{18}H_{34}O_2$	281.2497	-3.88
F10*	34.636	FA:S	$C_{18}H_{36}O_2$	283.2652	-3.33

C18 : 2 ,OH:Hydroxy octadecadienoic acid; Rn:ricinoleic acid; Ln:Linolenic acid; L:linoleic acid; O:oleic acid; P:palmitic acid; B:behenic acid; S:stearic acid.

* Structures confirmed by comparison with reference standards.

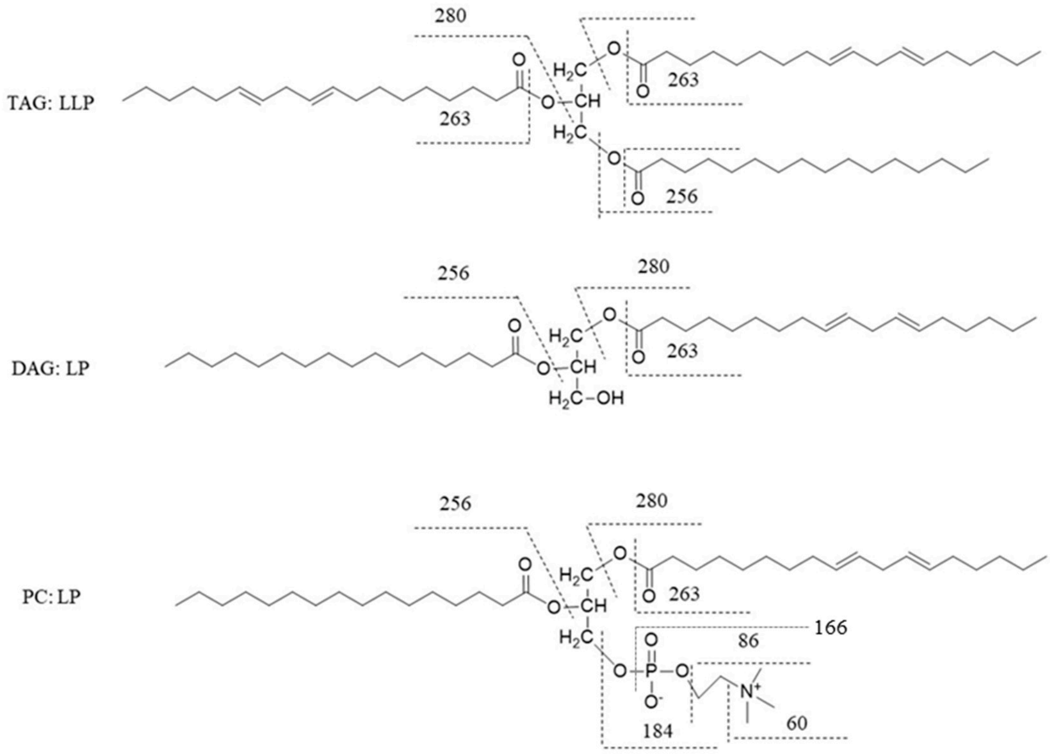

Figure S1 Fragmentation pattern of lipids compounds.

Table S7 The seven molecular species of TG contents from 77 batches of coix seed (from YC-01 to YC-77)

NO.	LLL(%)	LLP(%)	LLO(%)	POL(%)	OOL(%)	OOP(%)	OOO(%)	Total(%)
YC-01	0.15	0.21	0.48	0.35	0.54	0.30	0.56	2.59
YC-02	0.17	0.26	0.62	0.46	0.67	0.41	0.61	3.19
YC-03	0.18	0.28	0.65	0.47	0.70	0.41	0.64	3.33
YC-04	0.13	0.19	0.39	0.29	0.41	0.25	0.38	2.03
YC-05	0.31	0.49	1.35	0.89	1.46	0.69	1.25	6.44
YC-06	0.35	0.68	1.47	1.04	1.67	0.86	1.47	7.54
YC-07	0.34	0.54	1.41	1.02	1.47	0.80	1.25	6.83
YC-08	0.28	0.44	1.17	0.80	1.27	0.66	1.11	5.73
YC-09	0.20	0.30	0.77	0.54	0.79	0.49	0.78	3.86
YC-10	0.21	0.35	0.93	0.64	1.00	0.57	0.96	4.66
YC-11	0.36	0.48	1.15	0.80	1.18	0.60	0.95	5.52
YC-12	0.35	0.43	1.02	0.68	1.00	0.51	0.80	4.78

YC-13	0.40	0.51	1.21	0.77	1.17	0.51	0.85	5.42
YC-14	0.36	0.47	1.18	0.80	1.23	0.58	0.99	5.61
YC-15	0.36	0.46	1.15	0.79	1.25	0.61	1.02	5.64
YC-16	0.35	0.42	1.02	0.68	1.03	0.51	0.82	4.83
YC-17	0.36	0.46	1.15	0.77	1.18	0.58	0.96	5.47
YC-18	0.28	0.35	0.95	0.62	0.97	0.45	0.77	4.39
YC-19	0.24	0.28	0.78	0.52	0.81	0.37	0.60	3.60
YC-20	0.34	0.43	1.10	0.72	1.07	0.51	0.85	5.01
YC-21	0.28	0.38	1.01	0.65	1.05	0.47	0.83	4.67
YC-22	0.22	0.30	0.77	0.53	0.78	0.42	0.67	3.67
YC-23	0.23	0.33	0.82	0.57	0.84	0.44	0.70	3.92
YC-24	0.24	0.30	0.81	0.56	0.81	0.45	0.70	3.87
YC-25	0.15	0.19	0.42	0.28	0.42	0.23	0.36	2.04
YC-26	0.25	0.36	0.89	0.61	0.62	0.48	0.77	3.98
YC-27	0.14	0.18	0.42	0.29	0.46	0.21	0.33	2.03
YC-28	0.28	0.38	0.62	0.64	0.90	0.50	0.72	4.02
YC-29	0.28	0.37	0.92	0.63	0.90	0.49	0.75	4.33
YC-30	0.28	0.35	0.95	0.62	0.97	0.45	0.77	4.39
YC-31	0.32	0.45	1.20	0.82	1.23	0.63	1.04	5.68
YC-32	0.24	0.32	0.83	0.57	0.83	0.45	0.71	3.94
YC-33	0.26	0.35	0.90	0.68	0.97	0.50	0.81	4.47
YC-34	0.33	0.44	1.08	0.73	1.04	0.56	0.82	5.00
YC-35	0.33	0.42	1.10	0.70	1.05	0.51	0.81	4.92
YC-36	0.34	0.45	1.09	0.76	1.06	0.56	0.86	5.12
YC-37	0.28	0.36	1.01	0.58	0.96	0.42	0.69	4.30
YC-38	0.36	0.48	1.20	0.82	1.14	0.59	0.91	5.50
YC-39	0.24	0.33	0.78	0.55	0.79	0.42	0.65	3.76
YC-40	0.26	0.36	0.90	0.61	0.91	0.45	0.74	4.23
YC-41	0.23	0.26	0.68	0.46	0.76	0.29	0.48	3.16
YC-42	0.22	0.36	1.08	0.66	1.08	0.45	0.80	4.65
YC-43	0.26	0.36	1.09	0.54	1.09	0.32	0.81	4.48
YC-44	0.22	0.34	1.01	0.54	1.02	0.33	0.81	4.27
YC-45	0.30	0.30	0.95	0.47	0.97	0.28	0.77	4.05
YC-46	0.20	0.32	0.89	0.53	0.91	0.35	0.73	3.93
YC-47	0.22	0.34	1.02	0.57	1.05	0.36	0.85	4.40
YC-48	0.22	0.34	1.02	0.58	1.04	0.37	0.82	4.39
YC-49	0.25	0.39	1.19	0.66	1.20	0.42	0.94	5.05
YC-50	0.21	0.34	1.00	0.58	1.00	0.38	0.77	4.29
YC-51	0.16	0.27	0.69	0.48	0.72	0.36	0.61	3.29
YC-52	0.22	0.35	0.94	0.64	0.97	0.49	0.75	4.35
YC-53	0.23	0.41	1.14	0.82	1.25	0.67	1.11	5.63
YC-54	0.19	0.30	0.72	0.50	0.74	0.39	0.64	3.49
YC-55	0.22	0.37	0.95	0.69	1.06	0.59	1.00	4.89
YC-56	0.22	0.34	0.86	0.61	0.91	0.51	0.84	4.29

YC-57	0.32	0.47	1.16	0.78	1.08	0.60	0.94	5.35
YC-58	0.27	0.41	1.00	0.72	1.01	0.61	0.93	4.96
YC-59	0.30	0.45	1.12	0.78	1.12	0.64	0.99	5.39
YC-60	0.24	0.37	0.93	0.67	0.99	0.56	0.90	4.66
YC-61	0.21	0.34	0.80	0.57	0.84	0.48	0.77	4.00
YC-62	0.19	0.32	0.80	0.57	0.85	0.48	0.79	4.00
YC-63	0.34	0.47	1.24	0.81	1.15	0.61	0.84	5.47
YC-64	0.23	0.36	0.87	0.64	0.90	0.52	0.82	4.33
YC-65	0.22	0.35	0.87	0.62	0.91	0.52	0.83	4.32
YC-66	0.33	0.45	1.08	0.73	1.05	0.53	0.86	5.04
YC-67	0.30	0.39	1.01	0.63	0.99	0.45	0.77	4.54
YC-68	0.28	0.37	0.93	0.60	0.92	0.44	0.73	4.27
YC-69	0.34	0.43	1.12	0.72	1.07	0.51	0.84	5.03
YC-70	0.29	0.38	0.94	0.63	0.91	0.47	0.73	4.35
YC-71	0.27	0.36	0.95	0.58	0.87	0.41	0.64	4.06
YC-72	0.27	0.37	0.84	0.56	0.78	0.39	0.62	3.84
YC-73	0.34	0.45	1.11	0.74	1.06	0.53	0.85	5.08
YC-74	0.27	0.38	0.92	0.58	0.90	0.40	0.68	4.13
YC-75	0.32	0.42	1.00	0.67	0.98	0.49	0.78	4.67
YC-76	0.27	0.35	0.89	0.56	0.87	0.40	0.69	4.02
YC-77	0.38	0.51	1.19	0.84	1.16	0.65	0.97	5.70

Table S8 The rate of decline in the content of coix seed and coix seed powder.

Rate of decline (%)	coix seed			coix seed powder		
	YC-81	YC-82	YC-83	YC-81	YC-82	YC-83
0-3 month	2.03	4.86	8.29	21.72	20.79	19.60
3-6 month	12.96	1.61	7.86	8.81	6.90	8.90
6-9 month	22.16	24.49	20.27	38.70	34.49	35.05
9-12 month	55.00	47.06	54.71	75.27	73.60	75.20

Table S9 The calibration curves, linear range, limit of detection (LOD) and limit of quantification (LOQ) of the aflatoxins and zearalenone

Analytes	Linearity	Correlation coefficient (r)	Linear range （ng/mL）	LOQ （ng/mL）	LOD （ng/mL）
Aflatoxin G2	y = 103416x - 343.229	0.9998	0.12~3	0.07	0.013
Aflatoxin G1	y = 144101x + 9148.69	0.9994	0.05~10	0.03	0.008
Aflatoxin B2	y = 140006x - 420.935	0.9999	0.015~3	0.008	0.0017
Aflatoxin B1	y = 125602x + 5589.50	0.9995	0.05~10	0.04	0.006
Zearalenone	y = 264074x -111372	0.9995	1~150	0.095	0.015

The S10 Contents of aflatoxin and zearalenone in 24 batches of coix seed

No.	Aflatoxin G2 (μg/kg)	AflatoxinG1 (μg/kg)	AflatoxinB2 (μg/kg)	AflatoxinB1 (μg/kg)	Zearalenone (μg/kg)
YC-01	ND	ND	ND	ND	114.58
YC-02	ND	ND	ND	< 0.08	11.73
YC-03	ND	ND	ND	ND	2.7
YC-04	ND	ND	0.02	0.14	23.9
YC-05	ND	ND	ND	ND	26.41
YC-06	ND	ND	ND	ND	48.84
YC-07	ND	ND	ND	ND	10.92
YC-08	ND	ND	ND	ND	37.03
YC-31	ND	ND	ND	ND	20.88
YC-32	ND	ND	ND	ND	3.79
YC-33	ND	ND	ND	ND	2.31
YC-34	ND	ND	ND	< 0.08	27.2
YC-35	ND	ND	ND	ND	2.41

YC-36	ND	ND	ND	ND	4.08
YC-37	ND	ND	DT	< 0.08	9.44
YC-38	ND	ND	ND	ND	2.88
YC-58	ND	ND	< 0.03	ND	22.31
YC-59	ND	ND	ND	ND	24.32
YC-60	ND	ND	ND	ND	37.71
YC-61	ND	ND	ND	ND	9.44
YC-62	ND	ND	ND	ND	28.5
YC-63	ND	ND	ND	ND	16.99
YC-64	ND	ND	ND	ND	26.26
YC-65	ND	ND	ND	ND	2.34

[a]ND represents beyond the LOD

[b]DT represents between LOD and LOQ

[c] <0.03 or <0.08 represent between LOQ and lowest concentration of linearity
